# Factors associated to mortality in children with critical COVID-19 and multisystem inflammatory syndrome in a resource-poor setting

**DOI:** 10.1038/s41598-024-55065-x

**Published:** 2024-03-06

**Authors:** Emmerson C. F. de Farias, Manoel J. C. Pavão Junior, Susan C. D. de Sales, Luciana M. P. P. do Nascimento, Dalila C. A. Pavão, Anna P. S. Pinheiro, Andreza H. O. Pinheiro, Marília C. B. Alves, Kíssila M. M. M. Ferraro, Larisse F. Q. Aires, Luana G. Dias, Mayara M. M. Machado, Michaelle J. D. Serrão, Raphaella R. Gomes, Sara M. P. de Moraes, Gabriella M. G. Moura, Adriana M. B. de Sousa, Gabriela C. L. Pontes, Railana D. F. P. Carvalho, Cristiane T. C. Silva, Guilherme Lemes, Bruna da C. G. Diniz, Aurimery G. Chermont, Kellen F. S. de Almeida, Salma B. Saraty, Mary L. F. Maia, Miriam R. C. Lima, Patricia B. Carvalho, Renata de B. Braga, Kathia de O. Harada, Maria C. A. Justino, Gleice Clemente, Maria Teresa Terreri, Marta C. Monteiro

**Affiliations:** 1 Division of Pediatric Intensive Care, Department of Pediatrics, Fundação Santa Casa de Misericórdia do Pará, Belém, PA Brazil; 2 Medical School, Medical Science Institute, Federal University of Pará/UFPA, Belém, PA Brazil; 3 Division of Pediatric Intensive Care, Department of Pediatrics, Pronto Socorro Municipal Mário Pinotti’s Hospital, Belém, PA Brazil; 4 Division of Pediatric Intensive Care, Departament of Pediatrics, Fundação Hospital das Clínicas Gaspar Viana, Belém, PA Brazil; 5 Instituto Evandro Chagas, Virology Section, Health Surveillance Secretariat, Brazilian Ministry of Health, Ananindeua, PA Brazil; 6 Division of Pediatric Rheumatology, Department of Pediatrics, Universidade Federal de São Paulo, São Paulo, SP Brazil; 7 Pharmaceutical Science Post-Graduation Program and Neuroscience and Cell Biology Graduate Program, Health Science Institute, Federal University of Pará/UFPA, Belém, PA Brazil; 8 Department of Pediatric Critical Care, Fundação Santa Casa de Misericórdia do Pará, 7th Floor, St. Bernal do Couto, 988 - Umarizal, Belém, PA 66055-080 Brazil

**Keywords:** SARS-CoV-2 virus, Intensive Care Unit, Pediatric, Child health, Risk factor, Mortality, Paediatric research, Viral infection, Medical research, Infectious diseases

## Abstract

SARS-CoV-2 infection in children is usually asymptomatic/mild. However, some patients may develop critical forms. We aimed to describe characteristics and evaluate the factors associated to in-hospital mortality of patients with critical COVID-19/MIS-C in the Amazonian region. This multicenter prospective cohort included critically ill children (1 mo–18 years old), with confirmed COVID-19/MIS-C admitted to 3 tertiary Pediatric Intensive Care Units (PICU) in the Brazilian Amazon, between April/2020 and May/2023. The main outcome was in-hospital mortality and were evaluated using a multivariable Cox proportional regression. We adjusted the model for pediatric risk of mortality score version IV (PRISMIV) score and age/comorbidity. 266 patients were assessed with 187 in the severe COVID-19 group, 79 included in the MIS-C group. In the severe COVID-19 group 108 (57.8%) were male, median age was 23 months, 95 (50.8%) were up to 2 years of age. Forty-two (22.5%) patients in this group died during follow-up in a median time of 11 days (IQR, 2–28). In the MIS-C group, 56 (70.9%) were male, median age was 23 months and median follow-up was 162 days (range, 3–202). Death occurred in 17 (21.5%) patients with a median death time of 7 (IQR, 4–13) days. The mortality was associated with higher levels of Vasoactive Inotropic-Score (VIS), presence of acute respiratory distress syndrome (ARDS), higher levels of Erythrocyte Sedimentation Rate, (ESR) and thrombocytopenia. Critically ill patients with severe COVID-19 and MIS-C from the Brazilian Amazon showed a high mortality rate, within 12 days of hospitalization.

## Introduction

The novel coronavirus disease 2019 (COVID-19) pandemic caused by severe acute respiratory syndrome-coronavirus-2 (SARS-CoV-2) has affected over 100 million people worldwide, overwhelmed the health care system, and caused significant morbidity and mortality. In comparison to adults, children are less frequently affected, and most of them present with mild symptoms and better prognosis^1,2^. In hospitalized children, the presence of underlying medical conditions and age under one year old increases the risk of critical illness related to SARS-CoV-2, such as severe COVID-19 and multisystem inflammatory syndrome in children (MIS-C)^1–3^.

Several studies have been performed in high income countries, demonstrating characteristics of children with severe COVID-19/MIS-C requiring admission to Pediatric Intensive Care Units (PICU). Data regarding characteristics of critically ill children with COVID-19/MIS-C, and unfavorable prognosis, in resource-restricted regions, is lacking. Therefore, the risk factors for worse outcomes of COVID-19 in these studies may not be entirely applicable to other geographic regions or to less developed countries, which limits the extrapolation of the results^1–5^.

The Brazilian Amazonian region is recognized for economic, political, social, and environmental disparities, and is characterized by a vast territorial area which may result in lower quality of healthcare and limited accessibility to health services. However, the metropolitan region of Belém has PICUs with healthcare staff trained to care for critically ill children, adequate numbers of staff, and rapid access to necessary medications, supplies and equipment. The health disparities continue to exist in this region as a major issue that adversely affects groups of people who have systematically experienced greater obstacles to health care which result in an increase in health inequalities.

Given the genetic, socioeconomic conditions and healthcare access in the Amazonian region, critical COVID-19/MIS-C may exhibit different outcomes, including severity, duration, and sequelae compared to what has been described in other parts of the world^4–7^. We aimed to describe characteristics and evaluate the independent factors associated to in-hospital mortality of patients with critical COVID-19/MIS-C in the Amazonian region.

## Materials and methods

### Study design and participants

This multicenter prospective study included critically ill children between 1 month and 18 years of age, with confirmed critical COVID-19/MIS-C, admitted to PICU, from three tertiary Hospitals in the Brazilian Amazon, between April 2020 and May 2023. Patients with other causes of infection with similar presentations and absence of criteria for PICU were excluded. The study was approved by the institutional review board of the coordinating center (the other centers were co-participants). All participants and their legal guardians provided written informed assent and/or consent.

All children admitted to this study underwent diagnostic tests for the presence of SARS-CoV-2. SARS-CoV-2 infection was diagnosed either by nasopharyngeal swab RT-PCR and/or rapid antibody test for SARS-CoV-2, or serological test as recommended. Patients referred from outside facilities had previously had a mandatory test and even so, at the time of admission, a new exam was performed. Included children were separated into 2 groups: severe COVID-19 and critical MIS-C. Each group was split into two subgroups, based on in-hospital mortality at 28th day: survival and non-survival. All outcomes and the worst features were recorded and compared between the 2 groups.

### Data collection

The patients included in the study were prospectively assessed from date of hospital admission until discharge or death. The PICU admission criteria were the same in the three hospitals as there were specific guidelines and all of them are references in pediatric intensive care in the state. There was no uniform management protocol formulated by the researchers, however, in all units, the management protocol of pediatric patients with severe COVID-19/MIS-C was based on the recommendations of the Health Ministry. All parameters were collected and processed at the participating medical centers using standardized data collection forms, and all involved institutions used the same laboratory network for the analyses. In cases of readmission to PICU, only the first hospitalization was considered.

Data included demographic information, clinical, therapeutic, and outcomes. Laboratory and ventilatory parameters were evaluated on the first day of hospitalization. Presenting symptoms, respiratory support requirements, use of vasoactive medications and laboratory parameters, including inflammation markers (erythrocyte sedimentation rate—[ESR], C-reactive protein [CRP], d-dimer, fibrinogen, ferritin and triglycerides), and cardiac function (troponin T and creatine phosphokinase MB fraction [CPK-MB]) were collected. In addition, mortality and severity scores^8,9^, treatment, and unfavorable outcomes, were also assessed.

Critical disease included respiratory failure requiring invasive mechanical ventilation (IMV), acute respiratory distress syndrome (ARDS), shock, systemic inflammatory response syndrome, and/or multiorgan failure with at least one organ involvement. Multiple organ dysfunctions (MOD) were defined by the current criteria^10^. Severe COVID-19 was defined by the presence of positive real-time reverse transcription-polymerase chain reaction (RT-PCR), or antigen, and at least one organ dysfunction involvement, according to the current criteria^10,11^. MIS-C was defined according to World Health Organization (WHO) criteria^11^. All patients admitted to the units underwent the test before admission and were positive for antigen test and/or RT-PCR test. To the patients who had MIS-C criteria a serological test was added, as well in severe COVID-19 patients when the clinical presentation was similar to MIS-C.

Chest computed tomography and echocardiographic findings were recorded. Echocardiography was performed in patients with features of shock, unexplained tachycardia, and symptoms suggestive of MIS-C. Myocardial dysfunction was defined by the presence of global or segmental contractibility alterations, ventricular dilation, ejection fraction less than 55% measured by modified Simpson’s method, and troponin levels above the reference value^10–12^.

Treatment received by the patients such as: type of highest respiratory support, the number of ventilator days in those requiring invasive ventilation, highest Vasoactive Inotropic-Score (VIS)^13^ and current drug treatment (intravenous methylprednisolone pulse therapy, intravenous immunoglobulin, and low molecular heparin), were also analyzed. A standard ventilatory protocol was used in this study^14^. Septic shock related to COVID-19, ARDS, and acute kidney injury, were defined and managed as per the Surviving Sepsis Campaign International Guidelines for the Management of Septic Shock in children^12^, Pediatric Acute Lung Injury Consensus Conference (PALICC) definition^15^, and kidney disease: Improving Global Outcome classification system (KDIGO) guidelines^16^, respectively. We defined a SARS-CoV-2 infection associated pediatric death as death occurring in a patient with confirmed severe COVID-19/MIS-C who died after PICU admission. The admission criteria for PICU, was determined by the finding of multiorgan failure with at least one organ involvement which requires monitoring in the PICU, or diagnosis of acute respiratory syndrome or respiratory failure, circulatory shock of any kind, encephalopathy, hepatic or gastrointestinal dysfunction, myocardial dysfunction or heart failure, coagulation disorders, and acute kidney injury^10,12, 15, 16^. The primary outcome of interest was in-hospital mortality at 28th day.

### Statistical analysis

Cohort characteristics were summarized using median (interquartile range) for continuous variables, and frequencies (%) for categorical variables. Comparisons between groups were made using χ^2^ or Fisher exact tests for categorical variables, and Mann–Whitney U was used for continuous data. Holm-Bonferroni correction for multiple comparisons was employed, adjusting the significance level when patients were compared in two groups (survival and non-survival). Survival rates in days, were calculated, and Kaplan–Meier curves were plotted for categorical variables or using the method of fractional polynomials to access the scale of continuous variables. To determine risk factors for survival we performed univariate and multivariate analyses using continuous variables for the Cox regression “time-to-first-event” analysis. In the regression model we adjusted for a risk of mortality using pediatric risk of mortality score version IV (PRISMIV) score, age under one year old, and presence of comorbidity simultaneously. For these analyses the adopted significance level was set at two-sided p < 0.05. Data analyses were conducted using SPSS (Statistical Package for the Social Sciences, Chicago, IL) version 27.0.

### Ethics statement

The authors are accountable for all aspects of the work in ensuring that questions related to the accuracy or integrity of any part of the work are appropriately investigated and resolved. The study was conducted in accordance with the Declaration of Helsinki. The study was approved by the institutional review board of the coordinating center (the other centers were co-participants). The parents or guardians of the children, or, when applicable, the children themselves, provided written informed consent before being included in the study. Besides, confidentiality was maintained by keeping privacy at all levels of the study. The study was approved by the Research Ethics Committee of FSCMPA under number 0361/2017, opinion nº 4.060.894, CAAE 31513320.0.0000.5171.

### Consent to participate

Written informed consent was obtained from the parents.

## Results

### Description of selection criteria for the Cohort

**Figure 1:**
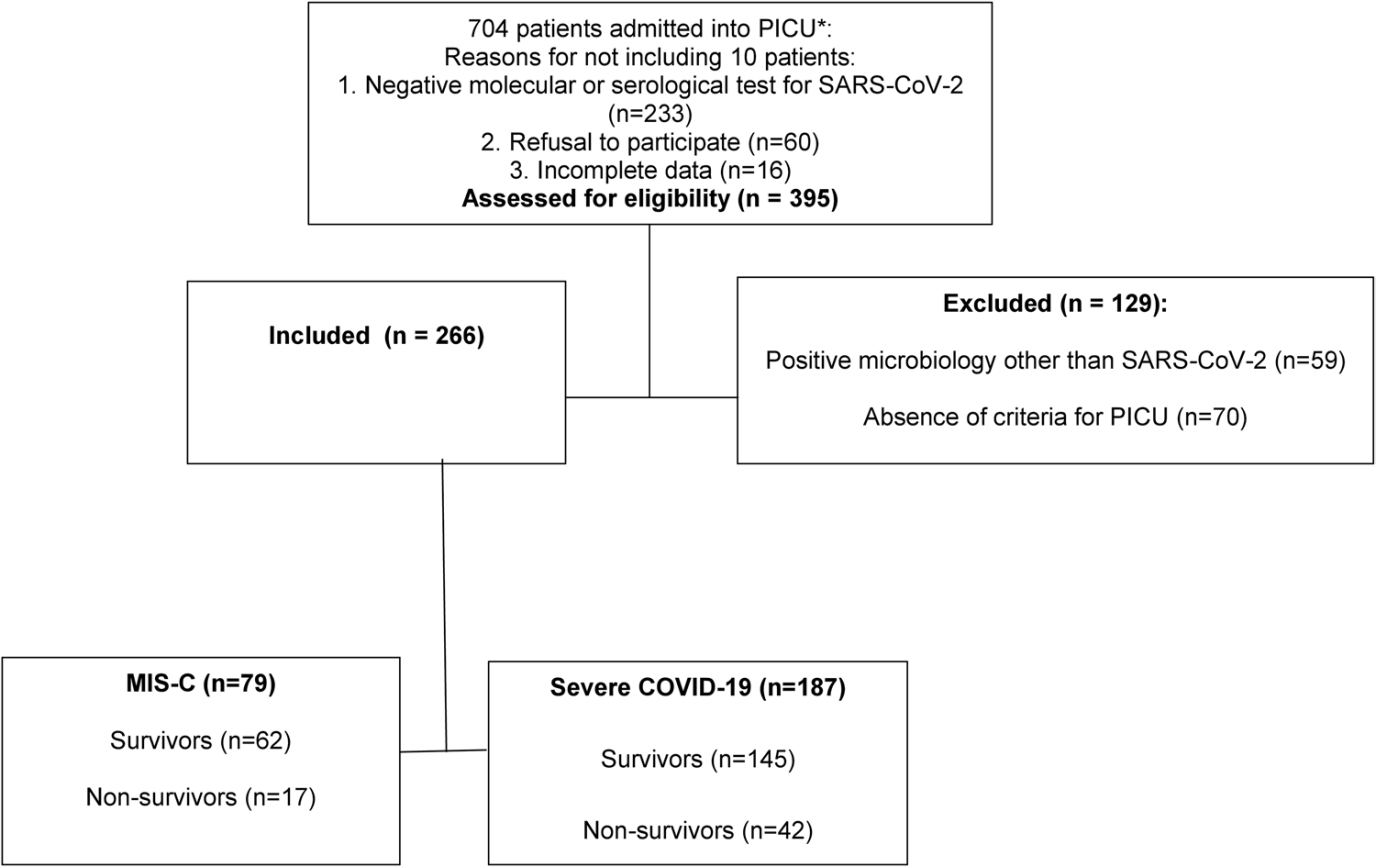
Flowchart describing the study design and selection of participants. PICU, Pediatric Intensive care unit.

In Brazil from April 2020 to June 2023, 2055 patients under 18 years old presented with confirmed MIS-C, with 140 (6.8%) deaths, while 59,059 patients under 18 years old presented with confirmed severe COVID-19, and were hospitalized, with 3571 (6.1%) deaths^17–20^. The vaccination coverage was around 7% in children under 2 years of age^20^. From a population of 704 patients admitted to PICUs in Belém, state of Pará, Brazil, with suspected SARS-CoV-2 infection, from April 01, 2020, to May 31, 2023, 204 patients were excluded due to the following reasons: negative molecular or serological test for SARS-CoV-2 (n = 233), absence of criteria for PICU (n = 70), and/or positive microbiology other than SARS-CoV-2 (n = 59); 7 were positive for other respiratory viruses, 39 were positive for bacteria, and 13 were positive for fungi. Data from 266 patients (median age, 33 [IQR, 9–36] months) was analyzed. The cohort was composed of 190 (71.4%) patients from the FSCMPA’s PICU, 60 (22.6%) patients from Hospital Mario Pinnoti’s PICU, and 16 (6%) from Fundacao Hospital de Clínicas’ Gaspar Viana. The mortality rate was 22.2% (59 patients). varying between 6.3 and 26.3% among the three units (Fig. 1).

### MIS-C group

The MIS-C group had 79 patients included, 56 (70.9%) were male, 40 (50.6%) were Brazilian pardo, with 17 (21.5%) non survival and 62 (78.5%) survival. The median age in the non-survival group was 17 months (IQR:5–33), 10 (58.8%) patients were Brazilian pardo and 15 (88.2%) were males. The main clinical presentation of MIS-C was Shock syndrome associated to Kawasaki disease, with 7 (41.2%) non-survival. Comorbidities were more frequent in the non-survival MIS-C group (48 [77.4%] vs. 16 [94.1%]), and also the need for IMV (17 [100%] vs. 38 [61.3%], p < 0.001). Myocardial dysfunctions were more pronounced in non-survival who clinically presented with cardiogenic shock (13 [76.5%] vs 20 [42.6%], p = 0.023) and higher VIS levels (median 163, IQR121-176, vs 12, IQR0-71). In MIS-C patients the Compliance Respiratory system (CRS) and driving pressure was not different and improved in a similar degree in both groups, survival (CRS median 0.6 mL/cmH_2_O/kg vs. 0.89 mL/cmH_2_O/kg, DP median 10 cmH_2_O vs. 9 cmH_2_O) and non-survival (median 0.67 mL/cmH_2_O/kg vs. 0.84 mL/cmH_2_O/kg, DP median 16 cmH_2_O vs. 17 cmH_2_O), with no statistical significance, with Wilcoxon test p = 0.135 and p = 0.34 for CRS and DP, respectively. The increase of ventilatory ratio^21,22^ in the MIS-C patients was performed to maintain the PCO_2_ levels according to the Tidal volume values. Some ventilatory settings and laboratory parameters were also associated with deaths, such as higher drive pressure levels, and worse hypoxemia at admission, and after 48 h, as shown in Table 1.

**Table 1:** Baseline characteristics of patients with critical MIS-C, according to in-hospital mortality.

Characteristics of patients with critical MIS-C	Survival (n = 62)	Non-survival (n = 17)	All patients (n = 79)	p value*
Epidemiological and demographics				
Age in months median (IQR)	28 (5–119)	17 (5–33)	23 (5–112)	0.126
Children under 2 years old n (%)	29 (46.8)	11 (64.7)	40 (50.6)	0.274
Sex: male n (%)	41 (66.1)	15 (88.2)	56 (70.9)	0.129
Ethnicity: Brazilian pardo n (%)	30 (48.4)	10 (58.8)	40 (50.6)	0.585
Pediatric complex chronic conditions n (%)	48 (77.4)	16 (94.1)	64 (81.0)	0.785
Comorbidities				
Neurologic and neuromuscular diseases	13 (27.1)	6(37.5)	19 (29.7)	0.756
Gastrointestinal diseases	9 (18.8)	4 (25)	13 (20.3)	1.000
Respiratory diseases	8 (16.7)	3 (18.8)	11 (17.2)	1.000
Time from exposure to symptom onset in days, median (IQR)	14 (11–21)	14 (9–25)	14 (11–23)	0.905
Duration of fever in days, median (IQR)	11 (7–13)	8 (5–12)	10 (6–12)	0.174
SARS-CoV-2 infection confirmed tests n (%)				
IgG	20/124 (16.1)**	3/34 (8.8)**	23/158 (14.6)**	0.412
Antigen test	42/124 (33.9)	14/34 (41.2)	56/158 (35.4)	0.431
RT-PCR test	12/124 (9.7)	4/34 (11.8)	16/158 (10.1)	0.75
BMI-for-age Z-scores > 5 years old: moderately underweight n (%)	11 (17.7)	4 (23.5)	15 (19.0)	1.000
BMI-for-age Z-scores < 5 years old: Normal weight n (%)	4 (6.5)	2 (11.8)	6 (7.6)	0.144
Clinical				
MIS-C clinical form: Shock syndrome Kawasaki disease n (%)	23 (37.1)	7 (41.2)	30 (38.0)	0.783
Lymphadenopathy n (%)	37 (59.7)	10 (58.8)	47 (59.5)	1.000
Severe dyspnea n (%)	17 (34.7)	12 (75)	29 (44.6)	0.008
Gastrointestinal symptoms n (%)	40 (64.5)	14 (82.4)	54 (68.4)	0.240
Pneumonia n (%)	35 (56.5)	13 (76.5)	48 (60.8)	0.168
Exanthema n (%)	40 (64.5)	15 (88.2)	55 (69.6)	0.078
Cardiogenic Shock n (%)	20 (42.6)	13 (76.5)	33 (51.6)	0.023
VIS median (IQR)	12 (0–71)	163 (121–176)	39 (0–91)	** < 0.001**
Oxygen therapy modalities n (%)				
Oxygen low interfaces	11 (17.7)	0 (0)	11 (13.9)	0.108
Invasive mechanical ventilation	38 (61.3)	17 (100)	55 (69.6)	** < 0.001**
Non-invasive mechanical ventilation	13 (21.0)	0 (0)	13 (16.5)	0.059
Ventilator weaning success at first attempt n (%)	36 (94.7)	5 (5.9)	41 (74.5)	** < 0.001**
Tracheostomy tube use n (%)	2 (5.3)	1 (5.9)	3 (5.5)	1.000
ADRS n (%)	23 (37.1)	17 (100)	40 (50.6)	** < 0.001**
Severe ARDS n (%)	5 (8.1)	10 (58.8)	15 (19)	0.004
MOD > 2 organ involvement n (%)	41 (66.1)	17 (100)	58 (73.4)	0.004
Renal replacement therapy n (%)	8 (12.9)	1 (5.9)	9 (11.4)	0.675
KDIGO stage 1, n (%)	20 (32.3)	16 (94.1)	36 (45.6)	** < 0.001**
Treatment n (%)				
Low molecular weight heparin therapy	33 (53.2)	16 (94.1)	49 (62.0)	0.003
Methylprednisolone pulse therapy	6 (9.7)	13 (76.5)	19 924.1)	0.002
Intravenous immunoglobulin therapy	37 (59.7)	17 (100)	54 (68.4)	0.002
Outcomes median (IQR)				
Length of stay at hospital, in days	15 (10–18)	11 (6–14)	14 (9–18)	0.105
Length of stay at PICU, in days	6 (4–8)	11 (6–14)	6 (4–9)	0.01
Mechanical ventilation time, in days	4 (3–6)	11 (6–14)	4 (3–9)	** < 0.001**
Ventilator free days at 28^th^	24 (22–25)	0 (0–1)	22 (1–25)	** < 0.001**
PRISMIV%	1.5 (1.2–2.8)	5.9 (1.9–32)	2.8 (1.5–26.4)	0.009
PELOD-2	9.5 (5–20)	15 (3–21)	11 (4–20)	0.9
Ventilatory settings and Respiratory mechanics median (IQR) Day 1				
Tidal volume in ml/kg	6.3 (5.4–7.3)	9.9 (9.3–10.6)	6.9 (5.6–9.5)	** < 0.001**
Respiratory rate *breaths/min*	24 (23–29)	25 (22–30)	26 (23–30)	0.813
Peep-peak paw in cmH_2_O	9 (7–10)	22 (18–24)	17 (10–23)	0.001
Driving pressure in cmH_2_O	9 (8–11)	17 (16–18)	11 (9–16)	** < 0.001**
Compliance respiratory system in mL/cmH_2_O/kg	0.6 (0.59–0.89)	0.67 (0.42–0.99)	0.64 (0.56–0.97)	0.91
Ventilatory ratio^a^	0.68 (0.48–1.61)	0.76 (0.62–1.13)	0.74 (0.53–1.3)	0.698
Laboratorial features median (IQR) Day 1				
Oxygenation index	5.3 (2.5–10)	10.6 (8.9–15.6)	8.4 (3.5–12.3)	** < 0.001**
PaO_2_/FiO_2_ ratio	238 (160–367)	159 (111–181)	204 (141–358)	** < 0.001**
pO_2_ (mmHg)	125.5 (72.4–180)	128 (73–151)	128 (72.4–179)	0.456
pCO_2_ (mmHg)	37.6 (28.6–55.9)	37 (29.4–50.6)	37.1 (28.8–55.9)	0.952
Sodium bicarbonate (mmol/L)	20.9 (18.2–23.7)	15.7 (13.8–22.9)	20.7 (16.3–23.7)	0.043
Anion gap (mmol/L)	7.7 (12.2–16.9)	18.2 (10.4–24.9)	16.9 (9.5–21.4)	0.016
Lactate (mmol/L)	2.3 (1.7–2.9)	2.7 (2–2.9)	2.3 (1.7–2.9)	0.962
Platelets count/mm^3^	194,000 (120,000–352,000)	28,000 (19,700–31,300)	152,000 (45,300–308,000)	** < 0.001**
Lymphocytes count/mm^3^	1,352 (1,038–1,998)	1,033 (774–1,428)	1,270 (978–1,870)	0.023
INR	1.2 (1.0–1.5)	1.2 (1.0–1.7)	1.2 (1–1.5)	0.440
d-Dimer (ng/dL)	1,917 (959–5,009)	1,606 (1,209–2,938)	1,789 (959–4,254)	0.872
Fibrinogen (mg/dL)	288 (145–453)	226 (160–254)	269 (145–385)	0.113
C-reactive protein(mg/dL)	34 (14,6–80,8)	77.6(34.5–88.7)	40.7 (16.2–85)	0,09
Erythrocyte sedimentation rate (mm/h)	12 (8–16)	45 (35–54)	14 (9–22)	** < 0.001**
Ferritin (ng/mL)	548 (291–597)	582 (482–1,112)	550 (298–616)	0.018
Urea (mg/dL)	26 (17–46)	22 (19–38)	25 (17–46)	0.441
Creatinine (mg/dL)	0.4 (0.3–0.9)	0.4 (0.3–0.4)	0.4 (0.3–0.8)	0.480
AST (IU/L)	45 (35–75)	50 (38–64)	46 (35–75)	0.527
ALT (IU/L)	35 (17–52)	38 (17–60)	35 (17–52)	0.716
Albumin (g/dL)	2.7 (2.3–3.8)	2.9 (2.5–3.5)	2.7 (2.3–3.8)	0.810
Troponin I (ng/L)	0.124 (0.01–1.35)	4.57 (2.1–11.31)	0.27 (0.03–2.8)	** < 0.001**
Ventilatory settings and Respiratory mechanics median (IQR) Day 2				
Tidal volume in ml/kg	5.8 (5.2–7.4)	9.7 (9–10)	7.2 (5.5–9.6)	** < 0.001**
Respiratory rate *breaths/min*	27 (23–29)	28 (22–30)	28 (23–30)	0.813
Peep- Peak Paw in cmH_2_O	10 (7–11)	19 (17–23)	17(10–22)	0.001
Driving pressure in cmH_2_O	10 (9–12)	16 (15–17)	12 (9–16)	** < 0.001**
Compliance respiratory system in mL/cmH_2_O/kg	0.89 (0.6–1.13)	0.84 (0.51–1.12)	0.87 (0.57–1.12)	0.68
Ventilatory ratio^a^	0.86 (0.6–1.08)	1.06 (0.88–2.2)	1.0 (0.69–1.86)	0.126
Laboratorial features median (IQR) Day 2				
Oxygenation index	4.1 (2.3–8.1)	10.9 (4.9–13.9)	6.5 (3.3–10.9)	0.006
PaO_2_/FiO_2_ ratio	348 (183–450)	151 (102–242)	257 (150–425)	0.001
pO_2_ (mmHg)	124 (58–157)	106 (59–131)	111 (55–147.5)	0.116
pCO_2_ (mmHg)	42 (34–65)	46 (42–49)	45 (34–63)	0.479
Sodium bicarbonate (mmol/L)	23 (19.4–27)	18.1 (15.8–20.3)	21 (17.7–25.6)	0,002
Anion gap (mmol/L)	11.7 (5.2–20.7)	15.3 (10.4–23.8)	13.3 (6.7–21.5)	0.07
Lactate (mmol/L)	1.4 (1–2.8)	3.5 (2.1–7.9)	1.9 (1.2–4)	0.059
Platelets count/mm^3^	265,800 (135,000–376,700)	301,000 (172,000–450,000)	279,000 (138,600–448,000)	0.761
Lymphocytes count/mm^3^	2,266 (1,346–5,000)	1,807 (1,102–2,934)	2,246 (1,248–4,525)	0.095
INR	1.1 (1–1.4)	1.11 (1.02–1.3)	1.1 (1.01–1.38)	0.2
d-Dimer (ng/dL)	1,002 (602 -1,795)	1,291 (690–3,861)	1,264 (609–3,861)	0.161
Fibrinogen (mg/dL)	203 (101–380)	170 (64–381)	203 (78–380)	0.605
C-reactive protein(mg/dL)	21 (7.8–41)	23 (7.5–67)	20 (7.3–60)	0.938
Erythrocyte sedimentation rate (mm/h)	20 (10–20)	56 (51–60)	20 (12–30)	** < 0.001**
Ferritin (ng/mL)	149 (94–851)	329 (169–580)	329 (132–580)	0,06
Urea (mg/dL)	15 (7–49)	34 (19–56)	31 (14–52)	0.037
Creatinine (mg/dL)	0.3 (0.25–0.5)	0.45 (0.25–0.6)	0.4 (0.25–0.6)	0.417
AST (IU/L)	41 (33–80)	53 (33–62)	49 (33–70)	0.605
ALT (IU/L)	31 (16–55)	32 (17–60)	31 (16–56)	0.543
Albumin (g/dL)	2.7 (2.4–3.2)	2.8 (2.3–3.1)	2.8 (2.5–3.2)	0.272
Troponin I (ng/L)	0.1 (0.01–7.8)	0.2 (0,02–19.5)	0.2 (0.012–13.4)	0.889

BMI, body mass index. VIS, vasoactive-inotropic score. ARDS, acute respiratory distress syndrome. MOD, Multiple organ dysfunction. KDIGO, Kidney Disease Improving Global Outcome classification system. Peep, Positive end-expiratory pressure. Paw, peak airway pressure. *The bold numbers refer to two-sided p value using Bonferroni correction (p < 0.001). Mechanical ventilation time, Ventilator weaning success at first attempt Oxygen inspired fraction %, was not used to avoid multicolinearity in the regression model. **All patients admitted to units underwent the test before admission and were positive for antigen test and/or RT-PCR test the patients who had MIS-C criteria we added serological test. ^a^Ventilatory ratio was defined as (minute ventilation × PaCO_2_)/(PBW × 100 × 37.5)^21^, because the predicted minute ventilation used in adults (100 mL/kg/min predicted body weight) does not apply over the age range of children and young adults, a pediatric predicted minute ventilation was used ^22^.

### Severe COVID-19 group

From 187 patients analyzed, 108 (57.8%) were male, median age was 23 months, 95 (50.8%) were up to 2 years of age, 78 were Brazilian pardo (41.7%), and 108 (57.8%) had at least one comorbidity. Forty-two (22.5%) patients died during follow-up, with a median time of 11 days (range, 2–28). Pneumonia was present in 33 (78.6%) in the non-survival group, with all deceased patients requiring IVM, only 15 (35.7%) had presented with severe ARDS. Some ventilatory settings and laboratory parameters were also associated with death, such as higher drive pressure levels, and worse hypoxemia. More pronounced lymphopenia at admission and higher levels of ESR at admission, as well as other parameters, were more frequent in non-survival compared to survival. We observed that on the second day of hospitalization, oxygen exchange was different between survival and non-survival (PaO_2_/FiO_2_ ratio, median 206 vs. 289, p = 0.018, Oxygen inspired fraction %, median 60 vs. 40, p = 0.57, and higher driving pressure levels median 10 vs. 8 cmH_2_O, p < 0.001), in addition to acute phase reactant (erythrocyte sedimentation rate in mm/h median, 44 vs. 15, p < 0.001) and pronounced coagulation disorders (median INR, 1.48 vs 1.26, p = 0.002). The increase of ventilatory ratio^21,22^ in the patients with severe COVID-19 patients was performed to maintain the PCO_2_ levels according to the Tidal volume values. In severe COVID-19 patients who died exhibited a significant deterioration of CRS between day 1 (median 0.78 mL/cmH_2_O/kg) and day 2 (median 0.60 mL/cmH_2_O/kg), as well as DP (day 1 median 13 cmH_2_O vs 10 cmH_2_O) despite having a higher value at the first day on IMV (Wilcoxon test p < 0.001 for both variables), as shown in Table 2.

**Table 2:** Baseline characteristics of patients with severe COVID-19, according to the in-hospital mortality group.

Characteristics of patients with severe COVID-19	Survival (n = 145)	Non-survival (n = 42)	All patients (n = 187)	p value*
Epidemiological and demographics				
Age in months median (IQR)	82 (12–132)	19 (6–82)	23 (6–81)	0.016
Children under 2 years old n (%)	80 (55.2)	15 (35.7)	95 (50.8)	0.026
Sex: male n (%)	78 (53.8)	30 (71.4)	108 (57.8)	0.041
Ethnicity: Brazilian pardo n (%)	68 (46.9)	10 (23.8)	78 (41.7)	0.007
Pediatric complex chronic conditions n (%)	76 (52.4)	32 (76.2)	108 (57.8)	0.006
Comorbitidies				
Neurologic and neuromuscular diseases	15 (19.7)	16 (50)	31(28.7)	0.003
Respiratory diseases	7 (9.2)	8 (25)	15 (13.9)	0.038
Prematurity and neonatal diseases	6 (7.9)	9 (28.1)	15 (13.9)	0.008
Time from exposure to symptom onset in days, median (IQR)	6 (5–9)	8 (5–15)	7 (5–9)	0.027
Duration of fever in days, median (IQR)	1 (1–4)	1 (1–4)	1 (1–4)	0.424
SARS-CoV-2 infection confirmed tests** n (%)				
RT-PCR	66/164 (40.2)	12/55 (21.8)	78/219 (35.6)	0.013
Antigen test	79/164 (48.2)	30/55 (54.5)	109/219 (49.8)	0.41
IgM test	11/164 (6.7)	2/55(3.6)	13/219 (5.9)	0.525
BMI-for-age Z-scores > 5 years old: Moderately underweight n (%)	6 (4.1)	10 (23.8)	16 (8.6)	0.006
BMI-for-age Z-scores < 5 years old: Moderately underweight n (%)	24 (16.6)	8 (19.0)	32 (17.1)	0.899
Clinical				
Lymphadenopathy n (%)	30 (20.7)	6 (14.3)	36 (19.3)	0.387
Dyspnea n (%)	102 (70.3)	42 (100)	144 (77.0)	** < 0.001**
Dyspnea classification n (%)	65 (63.7)	42 (100)	107 (74.3)	** < 0.001**
Gastrointestinal symptoms n (%)	55 (37.9)	13 (31.0)	68 (36.4)	0.406
Pneumonia n (%)	65 (44.8)	33 (78.6)	98 (52.4)	** < 0.001**
Exanthema n (%)	18 (12.4)	5 (11.9)	23 (12.3)	1.000
Cardiogenic Shock n (%)	28 (54.9)	23 (54.8)	51 (54.8)	1.000
VIS median (IQR)	0 (0–24)	120 (98–154)	0 (0–95)	** < 0.001**
Oxygen therapy modalities n (%)				
Oxygen low interfaces	77 (53.1)	0 (0)	77 (41.2)	** < 0.001**
Invasive mechanical ventilation	61 (42.1)	42 (100)	103 (55.1)	** < 0.001**
Non-invasive mechanical ventilation	7 (4.8)	0 (0)	7 (3.7)	0.209
Ventilator weaning success at first attempt n (%)	47 (77.0)	18 (42.9)	65 (63.1)	0.269
Tracheostomy tube use n (%)	8 (13.1)	3 (7.1)	11 (10.7)	0.712
ADRS n (%)	38 (26.2)	35 (83.3)	73 (39.0)	** < 0.001**
Severe ARDS n (%)	8 (5.5)	15 (35.7)	23 (12.3)	0.076
MOD > 2 organ involvement n (%)	26 (17.9)	14 (33.3)	40 (21.4)	0.036
Renal replacement therapy n (%)	13 (9.0)	6 (14.3)	19 (10.2)	0.383
KDIGO,stage 1, n (%)	60 (41.4)	26 (61.9)	86 (46.0)	0.025
Treatment n (%)				
Low molecular weight heparin therapy	51 (35.2)	9 (21.4)	60 (32.1)	0.132
Methylprednisolone pulse therapy	2 (1.4)	4 (9.5)	6 (3.2)	0.023
Intravenous immunoglobulin therapy	19 (13.1)	6 (14.3)	25 (13.4)	1.000
Outcomes median (IQR)				
Length of stay in hospital, in days	13 (9–18)	9 (5–15)	12 (8–17)	1.000
Length of stay at PICU, in days	3 (2–6)	6 (4–11)	4 (2–7)	** < 0.001**
Mechanical ventilation time, in days	4 (2–5)	6 (4–10)	4 (2–7)	** < 0.001**
Ventilator free days at 28^th^	24 (23–26)	0 (0–1)	21 (0–24)	** < 0.001**
PRISMIV%	3 (2–8)	8.6 (4–13)	4 (2–9)	** < 0.001**
PELOD-2	6 (3–12)	11 (2.8–15)	6 (3–12)	0.036
Ventilatory settings and respiratory mechanics median (IQR) Day 1				
Tidal volume in ml/kg	7 .1(6.2–8.3)	7.3 (6.1–8.2)	7.1 (6.1–8.2)	0.417
Respiratory rate *breaths/min*	20 (14–28)	25 (24–30)	25 (17–30)	0.064
Peep-peak paw in cmH_2_O	16 (15–18)	19 (17–22)	17 (15–19)	0.033
Driving pressure in cmH_2_O	10 (7–13)	13 (8–15)	11 (7–14)	0.015
Compliance respiratory system in mL/cmH_2_O/kg	0.88 (0.62–1.16)	0.78 (0.51–1.01)	0.66 (0.58–0.95)	0.035
Ventilatory ratio^a^	0.61 (0.5–0.84)	0.85 (0.64–1.12)	0.79 (0.59–1.08)	0.002
Laboratorial features median (IQR) Day 1				
PaO_2_/FiO_2_ ratio	287 (202–431)	160 (108–258)	268 (154–402)	** < 0.001**
pO_2_ (mmHg)	137 (83.4–183)	87.1 (73–124.3)	124.3 (75.7–180)	0.002
pCO_2_ (mmHg)	35.2 (30.2–44)	36.4 (32.1–43.8)	33.8 (31–42.3)	0.036
Sodium bicarbonate (mmol/L)	21 (18.2–25.5)	18.1 (15.4–21)	20 (16.8–24.5)	0.001
Anion gap (mmol/L)	13.3 (9.3–19.9)	18.2 (14.4–20.5)	14.2 (9.9–19.9)	0.007
Lactate (mmol/L)	1.7 (1.1–2.6)	1.8 (1.1–2.9)	1.7 (1.1–2.6)	0.821
Total leukocyte count/mm3	12,495 (9000–18,260)	8957 (6460–11,065)	11,400 (7569–17,1010)	** < 0.001**
Lymphocytes count/mm3	2780 (1518–4867)	1113 (855–1841)	2432 (1113–4554)	** < 0.001**
INR	1.21 (1.02–1.56)	1.5 (1.2–1.65)	1.25 (1.03–1.58)	0.011
d-Dimer (ng/dL)	609 (330–1,940)	516 (289–1,192)	575 (312–1,813)	0.05
Fibrinogen (mg/dL)	125 (102–174)	109 (92–134)	120 (100–173)	0.023
C-reactive protein(mg/dL)	6 (2–15.4)	2.3 (0.6 -9.4)	6 (1.3–14.1)	0.006
Erythrocyte sedimentation rate (mm/h)	12 (10–16)	17 (10–35)	14 (10–17)	** < 0.001**
Ferritin (ng/mL)	230 (142–452)	255 (224–385)	241 (144–452)	0.389
Urea (mg/dL)	22 (14–43)	19 (10–45)	22 (14–44)	0.404
Creatinine (mg/dL)	0,4 (0.2–0.6)	0.5 (0.3–0.7)	0.4 (0.2–0.6)	0.05
AST (IU/L)	39 (24–55)	32 (13–60)	37 (22–56)	0.255
ALT (IU/L)	33 (21–38)	35 (31–39)	34 (21–39)	0.126
Albumin (g/dL)	2.8 (2.5–3.4)	2.6 (2.1–3.1)	2.8 (2.3–3.4)	0.037
Troponin I (ng/L),	0.07 (0.012–0.189)	0.1 (0.031–0.188)	0.094 (0.02–0.189)	0.685
Ventilatory settings and respiratory mechanics median (IQR) Day 2				
Tidal volume in ml/kg	5.7 (4.1–7.4)	8.1 (6.6–9.8)	6.6 (4.1–8.2)	0.006
Respiratory rate *breaths/min*	22 (20–25)	24 (23–28)	24 (21–27)	0.285
Peep-peak paw in cmH_2_O	15 (14–21)	20 (19–23)	19 (15–21)	0.055
Driving pressure in cmH_2_O	8 (6–9)	10 (8–12)	8 (5–11)	** < 0.001**
Compliance respiratory system in mL/cmH_2_O/kg	0.81 (0.6–1.04)	0.6 (0.41–0.72)	0.88 (0.57–1.12)	0.001
Ventilatory ratio^a^	0.81 (0.61–0.93)	1.27 (0.77–1.73)	0.85 (0.66–1.28)	0.001
Laboratorial features median (IQR) Day 2				
PaO_2_/FiO_2_ ratio	289 (187–469)	206 (119–340)	274 (186–417)	0.018
pO_2_ (mmHg)	99 (62–141)	108 (71–139)	103 (63–141)	0.681
pCO_2_ (mmHg)	36 (30–42)	35 (27–42)	36 (29–42)	0.278
Sodium bicarbonate (mmol/L)	22.3 (18–24.8)	20.1 (15.5–22.4)	21.6 (17.7–24.6)	0.189
Anion gap (mmol/L)	14.2 (9.1–19.4)	15.4 (12.1–20.7)	14.2 (9.7–19.7)	0.256
Lactate (mmol/L)	1.5 (1–2.1)	1.3 (1–2.3)	1.4 (1–2.2)	0.365
Total leukocyte count/mm3	11,821 (8017–16,161)	11,649 (8069–18,327)	11,719 (8048–16,423)	0.267
Lymphocytes count/mm3	2710 (1122–4402)	2000 (1092–3122)	2640 (11,122–4149)	0.039
INR	1.26 (1.16–1.5)	1.48 (1.21–1.91)	1.27 (1.16–1.58)	0.002
d-Dimer (ng/dL)	510 (258–1371	1782 (483–5,178)	546 (258–1783)	0.154
Fibrinogen (mg/dL)	131 (93–269)	87 (64–270)	120 (86–269)	0.246
C-reactive protein(mg/dL)	6 (1.3–11)	6.3 (0.8–13.5)	6 (1.1–12)	0.67
Erythrocyte sedimentation rate (mm/h)	15 (10–18)	44 (39–77)	15 (10–22)	** < 0.001**
Ferritin (ng/mL)	199 (128–502)	372 (219–600)	221 (128–504)	0.473
Urea (mg/dL)	22 (11–43)	26 (9–85)	22 (11–44)	0.263
Creatinine (mg/dL)	0.5 (0.3–0.8)	0.5 (0.3–0.7)	0.5 (0.3–0.8)	0.804
AST (IU/L)	51 (36–94)	52 (36–75)	51.5 (36–84)	0.444
ALT (IU/L)	42 (21–65)	45 (26–50)	42 (23–65)	0.848
Albumin (g/dL)	3 (2.6–3.6)	2.9 (2.5–4)	3 (2.6–3.8)	0.447
Troponin I (ng/L),	0.046 (0.011–0.33)	0.048 (0.01–0.1)	0.47 (0.01–0.31)	0.013

BMI, body mass index. VIS, vasoactive-inotropic score. ARDS, acute respiratory distress syndrome. MOD, Multiple organ dysfunction. KDIGO, Kidney Disease Improving Global Outcome classification system. Peep, Positive end-expiratory pressure. Paw, peak airway pressure. *The bold numbers refer to two-sided p value using Bonferroni correction (p < 0.001). Mechanical ventilation time, oxygen low interfaces, dyspnea were not used to avoid multicollinearity in the regression model. **All patients admitted to units underwent the test before admission and were positive for antigen test and/or RT-PCR test the patients who had MIS-C criteria we added serological test. ^a^Ventilatory ratio was defined as (minute ventilation × PaCO_2_)/(PBW × 100 × 37.5)^21^ , because the predicted minute ventilation used in adults (100 mL/kg/min predicted body weight) does not apply over the age range of children and young adults, a pediatric predicted minute ventilation was used^22^.

### Univariate and multivariate analysis

After Cox regression multivariate analysis in the MIS-C group, the main factor associated with in-hospital mortality was platelets lower than 100,000 cel/mm^3^. Every decrease of 15,000 cel/mm^3^ platelets increases the risk by 10% (HR1.1, CI95%, 1.0–1.2, p < 0.001) and 40% (HR 1.4, CI95%, 1.1–1.6, p = 0.01) in adjusted models 1 and 2, respectively. In the severe COVID-19 group, VIS (each 5 change increase 30%, 3% and 5% for crude model, adjusted model1 and adjusted model 2, respectively) and ARDS (crude model HR 6.9, CI95%, 1.4–34.5, p = 0.018; adjusted model 1 and 2, HR, 6.4, CI95%, 1.31–31.5, p = 0.022 and HR, 13, CI95%, 2–83, p = 0.007, respectively) stood out as the main factors associated with in-hospital mortality, in models, as shown in Table 3.

**Table 3:** Cox regression analysis for associated factors to mortality at admission, according to the study groups.

MIS-C group	Crude model						Adjusted model 1*						Adjusted model 2*					
	Univariate			Multivariate			Univariate			Multivariate			Univariate			Multivariate		
Variable	HR	CI 95%	p value	HR	CI 95%	p value	HR	CI 95%	p value	HR	CI 95%	p value	HR	CI 95%	p value	HR	CI 95%	p value
Troponin I (ng/L) for 0.01 ng/L change for each	1.01	0.98–1.04	0.516	–	–	–	1.0	0.99–1.01	0.98	–	–	–	1.01	0.99–1.01	0.708	–	–	–
Platelets for 15,000 cel/mm^3^ change for each	1.02	0.99–1.2	**0.001**	1.01	0.99–1.03	**0.007**	1.11	1.0–1.22	** < 0.001**	1.12	1.02–1.23	**0.010**	1.3	1.1–1.5	0.001	1.4	1.1–1.6	**0.010**
ESR mm/h	1.09	1.05–1.2	** < 0.001**	1.3	1.03–1.5	**0.020**	1.1	1.06–1.17	** < 0.001**	1.2	0.99–1.5	0.057	**1.2**	**1.1–1.4**	** < 0.001**	1.2	1.01–1.5	**0.04**

Severe COVID-19 group	Crude model						Adjusted model 1*						Adjusted model 2*					
	Univariate			Multivariate			Univariate			Multivariate			Univariate			Multivariate		
Variable	HR	CI 95%	p value	HR	CI 95%	p value	HR	CI 95%	p value	HR	CI 95%	p value	HR	CI 95%	p value	HR	CI 95%	p value
Pneumonia	3.7	1.8–7.8	** < 0.001**	2.5	0.60–11.5	0.233	0.7	0.06–7.5	0.761	–	–	–	1.61	0.2–15	0.679	–	–	–
ARDS	10.4	4.6–23.4	** < 0.001**	6.9	1.4–34.5	**0.018**	38.4	8.6–73.2	** < 0.001**	6.4	1.31–31.5	**0.022**	37.1	7.8–76	< 0.001	13	2–83	**0.007**
VIS for 5 change each	1.03	1.01–1.04	** < 0.001**	1.3	1.2–1.4	** < 0.001**	1.02	1.01–1.05	** < 0.001**	1.03	1.02–1.04	** < 0.001**	1.03	1.02–1.04	< 0.001	1.05	1.03–1.06	** < 0.001**
Leukocyte/mm^3^ for 1000 cel/mm^3^ change each	1.01	0.98–1.02	0.238	–	–	–	1.02	1.01–1.03	0.179	–	–	–	0.98	0.95–1.01	0.505	–	–	–
ESR mm/h for 5 mm/h change each	1.08	1.04–1.11	** < 0.001**	1.05	0.98–1.12	0.214	1.07	1.04–1.11	** < 0.001**	1.06	1.0–1.2	**0.037**	1.08	1.05–1.2	< 0.001	1.06	1–1.4	0.073

ARDS, acute respiratory distress syndrome. VIS, Vasoactive-inotropic score. PIP, peak inspiratory pressure. ESR, Erythrocyte sedimentation rate. PRISMIV%, pediatric risk of mortality score version IV. *Adjusted model 1 and 2 for PRISMIV score > 9 and age under one year old/ presence of comorbidity, respectively. Lymphadenopathy, Dyspnea, Oxygen low interfaces, Invasive mechanical ventilation, Length of stay at PICU, in days, Mechanical ventilation time, in days, Lymphocytes count/mm^3^, Ventilator free days at 28th, and PaO_2_/FiO_2_ ratio did not show the proportionality of risks in the Kaplan Meyer curves in the severe COVID-19 group, while in the MIS-C group the variables: Peak inspiratory pressure, tidal volume, driving pressure, KDIGO stage 1, ARDS, VIS, Invasive mechanical ventilation, Ventilator weaning success at first attempt, Ventilator free days at 28th, Mechanical ventilation time, Oxygen inspired fraction, Positive end-expiratory pressure, PaO_2_/FiO_2_ ratio, also did not show the proportionality of risks in the Kaplan Meyer curves or high colinearity or worst result of fractional polynomials method.

The bold number refers to the two-sided p value (p < 0.05).

### Survival analysis

Critically ill patients with severe COVID-19 and MIS-C from the Brazilian Amazon presented a median time of 12 days of hospitalization until death.

### MIS-C group

After a median follow-up of 162 days (IQR, 156–168, range, 3–202), there were 17 deaths (24.3%) in the critical MIS-C group. The median time for death was 7 days (IQR, 4–13, range 2–40). The Kaplan–Meier survival plots for the statistical general mortality and risk factors in the critical MIS-C group are presented in Supplementary Information 1-Figure (A-B).

### Severe COVID-19 group

In the severe COVID-19 group the general mean survival time was 240.7 days (SD 9.04, CI95%, 222.9–258.4) with a median follow-up time of 161 days (IQR, 153–169, range, 2–307). There were 42 (22.5%) deaths and the median time until death was 11 days (IQR, 7–29, range, 3–55). The Kaplan–Meier survival plots for the statistical general mortality and risk factors in the severe COVID-19 patients are presented in Supplementary Information 2-Figure (A-D). Other data information about each patient is available in Supplementary Information 3-Table 1.

## Discussion

Our key findings suggest a higher mortality rate for COVID-19 (22.5%) and MIS-C (21.5%) patients compared to other pediatric studies from high-income countries (range 1–10%)^1–3, 5–7, 23–25^. It is worth noting that most of these studies also included non-critical patients, which makes an adequate comparison difficult, as our sample consisted only of critically ill patients. The mortality rate was high and was characterized by a significant disparity among the three PICUs. This could be due to the inequality in the number of patients attended in the three units.

The main factors associated with in-hospital mortality in this study were the presence of ARDS and cardiovascular dysfunction in the COVID-19 group, while in the MIS-C group an increase in inflammatory markers and thrombocytopenia stood out. These findings are associated with the greater risk factors for PICU admission among the pediatric population with COVID-19/MIS-C, as reported in previous studies^1,3, 5, 6, 20, 24, 26–29^, however, their presence would not be able to justify mortality exclusively, since in these other studies, the mortality rate was lower than that found in our study.

Based on this we hypothesize that our findings may arise from a complex interaction that emerged between social and clinical risk factors for fatal outcomes. Several studies^30–32^ have shown that the interactions between ethnicity, social inequality, and/or health access disparity, are strongly associated with the unfavorable outcomes in COVID-19. Among social factors, adult patients^29,30^ and children/adolescents^33,31^ from the poorest regions of the country, including those of Indigenous/Pardo Brazilian ethnicity, had a significantly higher risk of poor outcomes.

This study revealed a shorter time to death compared to other surveys. To the best of our knowledge, there are currently no reports regarding the time to death among the pediatric population. However, an adult cohort from a developed country revealed a longer time to death of between nine and 12 days^34^, which is similar to our findings. Most patients were severely ill and died during their initial days in hospital. As revealed in the ventilatory and laboratorial parameters on the first day of hospitalization, the patients arrived severely ill at the hospital, which could have been a contributor to the short time to death. The vast geographic region and scarcity of specialized centers may have contributed to longer time to access the health system, and consequently, the lower survival time.

Our findings indicate the presence of unfavorable outcomes in our cohort. We hypothesize that numerous biological factors could be determinants of this scenario, such as, high rate of comorbidity, ethnicity and genetic factors, presence of malnutrition, high severity of cases, among others. In addition, the influence of socioeconomic inequities, may coexist as confounding variables, however we were not able to provide this data. Health disparities favor illness, and consequently, worse outcomes for affected individuals or groups, which are reinforced by health inequities. The concept of structural vulnerability seeks to integrate all factors outside the clinical environment to more holistically contextualize the barriers and facilitators that individuals or groups may face in accessing healthcare, adhering to treatment protocols, and achieving better health outcomes. The factors involved in health disparities differ depending on where these disparities occur throughout the course of acute critical illness, and a clear understanding of where these disparities exist is critical to designing future interventions to improve outcomes for all patients^35,36^.

This study has some limitations. Firstly, there was selection bias in our cohort, since only patients with critical COVID-19/MIS-C and patients from low socio-economic status were included, although a specific socio-economic questionnaire was not performed. In addition to this, although the management of patients was based on Health Ministry recommendations for COVID-19/MIS-C, a uniform management protocol was not applied. It is important to report that the mortality rate was higher in 2020 than in 2021 in MIS-C and in severe COVID-19 groups and has progressively reduced: total of 43 deaths in 2020 (13 [30.2%] and 30 [69.8%] respectively), 7 deaths in 2021 (4 [57.2%] and 3 [42.8%], respectively), and 6 deaths in 2022 (0 [0%]) and 6 [100%], respectively), and finally 3 deaths in 2023 (0 [0%] and 3 [100%], respectively). There was also a change in the ventilatory management of patients with COVID-19 in 2021, based on evidence extrapolated from adults, with less frequent use of alveolar recruitment, and monitoring with bedside electrical impedance tomography to choose the best PEEP. In the case of MIS-C, the implementation of vasoactive support and immunomodulatory therapy was earlier.

An important strength of this study is that it contributes to better knowledge regarding risk factors for severe illness and mortality, which will allow the best prognosis for patients. Furthermore, our study is unique in terms of the large sample size of critical patients with laboratory-confirmed COVID-19, allowing good quality analysis. Another strength is the strict data collection and analysis, which was triple checked, in order to minimize mistakes. To the best of our knowledge, this is the largest sample from Brazil which comprehensively describes factors associated to in-hospital mortality in children with severe COVID-19/MIS-C.

In our study the risk of death at any time during follow-up was clearly higher for patients with ARDS and higher levels of VIS in the severe COVID-19 group, and higher ESR and thrombocytopenia for those who presented with MIS-C. It is urgent that we increase surveillance regarding critical forms of pediatric COVID-19 in poor regions. Further data is required to manage children with critical COVID-19/MIS-C, especially in limited-resource regions to provide high quality care for those patients with these life-threatening illnesses.

### Supplementary Information

Supplementary Information 1.

Supplementary Information 2.

Supplementary Information 3.

## Data Availability

Due to the privacy of patients, the data related to patients cannot be available for public access but can be obtained from the corresponding author (emmersonfariasbrandynew@gmail.com) on reasonable request approved by the institutional review board of all enrolled centers.

## Abbreviations

ARDS: Acute respiratory distress syndrome
COVID-19: Coronavirus disease 2019
CRP: C-reactive protein
CPK-MB: Creatine phosphokinase MB fraction
ESR: Erythrocyte sedimentation rate
IMV: Invasive mechanical ventilation
KDIGO: Kidney disease improving global outcome classification system
MIS-C: Multisystem inflammatory syndrome in children
MOD: Multiple organ dysfunction
OI: Oxygenation index
PALICC: Pediatric acute lung injury consensus conference
PICU: Pediatric Intensive Care Unit
SARS-CoV-2: Severe acute respiratory syndrome-coronavirus-2
VIS: Vasoactive inotropic-score
WHO: World Health Organization
