# Supplementary material for: Factors associated to mortality in children with critical COVID-19 and multisystem inflammatory syndrome in a resource-poor setting
Source: Sci Rep. 2024 Mar 6;14:5539. doi: 10.1038/s41598-024-55065-x (PMC10918095; doi:10.1038/s41598-024-55065-x)
Supplement: Supplementary file 1 — Supplementary Information 1. [file 41598_2024_55065_MOESM1_ESM.pdf]

Supplementary Information 1-Figure (A-B).

Factors associated to mortality in children with COVID-19 and Multisystem Inflammatory Syndrome in the PICU in a resource-poor setting

Authors

Emmerson C.F. de Farias[a], MD, Manoel J.C. Pavão Junior[a], MD, Susan C.D. de Sales[a], MD, Luciana M.P.P. do Nascimento[a], MD, Dalila C.A. Pavão[a], MD, Ana P.S. Pinheiro[a], MD, Andreza H.O. Pinheiro[a], MD, Marília C.B. Alves[a], MD, Kíssila M.M.M Ferraro[a], MD, Larisse F.Q. Aires[a], MD, Luana G. Dias[a], MD, Mayara M.M. Machado[a], MD, Michaelle J.D. Serrão[a], MD, Raphaella R. Gomes[a], MD, Sara M.P. de Moraes[a], MD, Gabriella M. Galvão[a], MD, Adriana MB de Sousa[a], MD, Gabriela C.L. Pontes[a], MD, Railana D.F.P. Carvalho[a], MD, Cristiane T.C. Silva[a], MD, Guilherme Lemes[a], MD, Bruna da C.G. Diniz[a], MD, Aurimery G. Chermont[a], Ph.D., Kellen F.S. de Almeida[a], Ph.D, Salma B.Saraty[b], Ph.D., Mary L.F. M.F. de Mello[b], MD, Miriam R.C. Lima[b], MD, Patricia B. Carvalho[c], MD, Renata de B. Braga[c], MD, Kathia de O. Harada[c], MD, Maria C.A. Justino[d], Ph.D, Gleice Clemente[e], Ph.D, Maria Teresa Terreri[e, #], Ph.D, Marta C. Monteiro, Ph.D[f, #].

[#] These authors have contributed equally to this work as co-senior authorship.

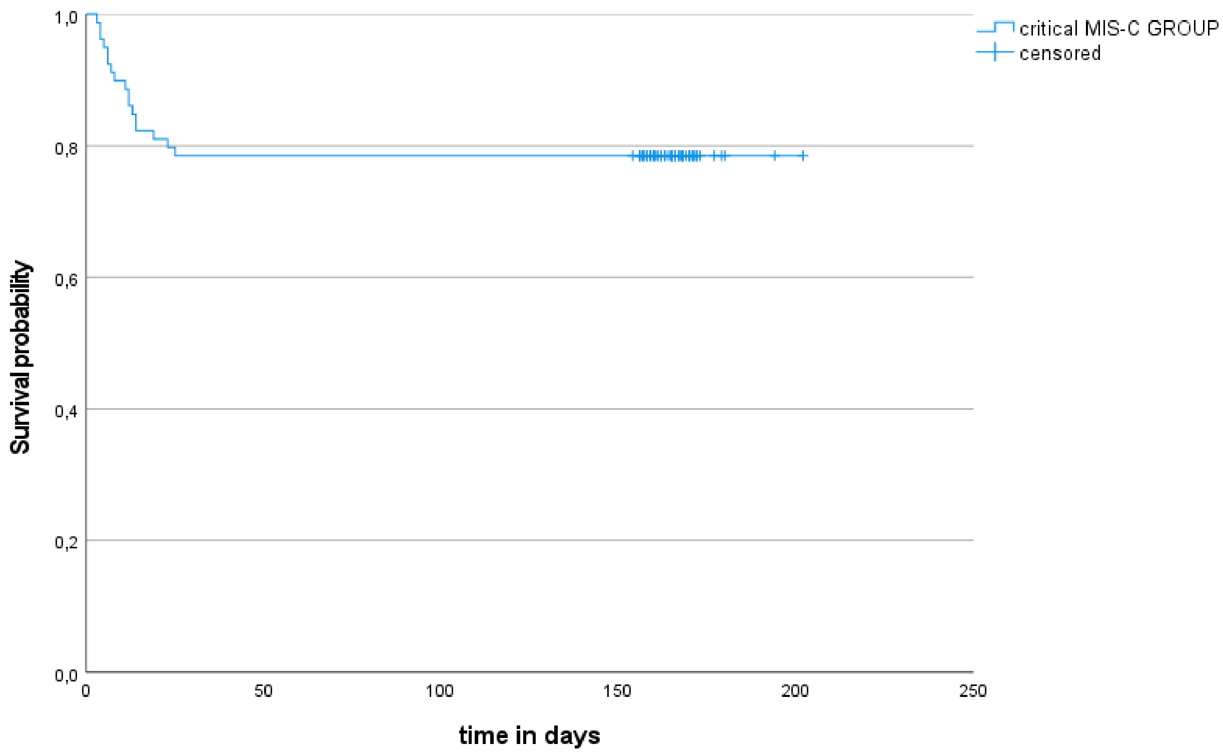

A

Number at risk by time in critical MIS-C group							
Number of cases	79	78	74	71	68	5	0
Time in days	0	3	6	9	12	173	202

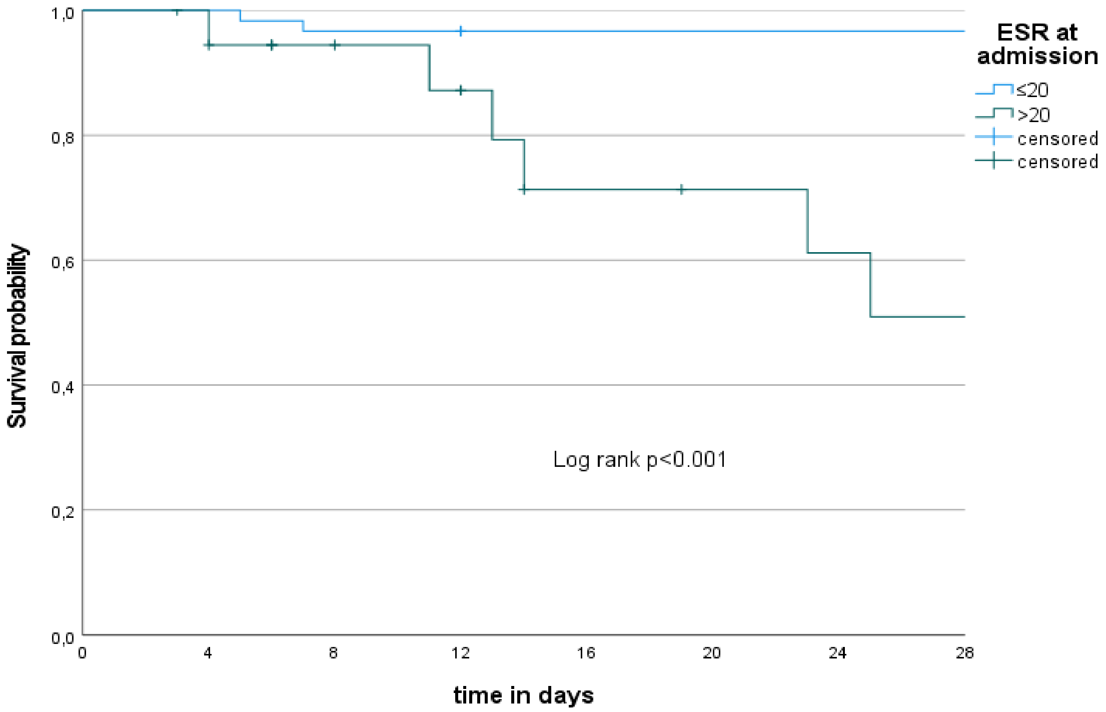

B

ESR mm/h	Number at risk by time in critical MIS-C adjusted model					
ESR≤20	60	60	59	58	56	56	13
ESR>20	19	18	14	13	7	5	0
Time in days	0	3	6	9	15	25	170
