# Supplementary material for: Factors associated to mortality in children with critical COVID-19 and multisystem inflammatory syndrome in a resource-poor setting
Source: Sci Rep. 2024 Mar 6;14:5539. doi: 10.1038/s41598-024-55065-x (PMC10918095; doi:10.1038/s41598-024-55065-x)
Supplement: Supplementary file 3 — Supplementary Information 3. [file 41598_2024_55065_MOESM3_ESM.pdf]

Supplementary Information 3- Table 1 – Characteristic of each patient of the Cohort

Factors associated to mortality in children with critical COVID-19 and Multisystem Inflammatory Syndrome in a resource-poor setting

Authors

Emmerson C.F. de Farias[a], MD, Manoel J.C. Pavão Junior[a], MD, Susan C.D. de Sales[a], MD, Luciana M.P.P. do Nascimento[a], MD, Dalila C.A. Pavão[a], MD, Ana P.S. Pinheiro[a], MD, Andreza H.O. Pinheiro[a], MD, Marília C.B. Alves[a], MD, Kíssila M.M.M Ferraro[a], MD, Larisse F.Q. Aires[a], MD, Luana G. Dias[a], MD, Mayara M.M. Machado[a], MD, Michaelle J.D. Serrão[a], MD, Raphaella R. Gomes[a], MD, Sara M.P. de Moraes[a], MD, Gabriella M. Galvão[a], MD, Adriana MB de Sousa[a], MD, Gabriela C.L. Pontes[a], MD, Railana D.F.P. Carvalho[a], MD, Cristiane T.C. Silva[a], MD, Guilherme Lemes[a], MD, Bruna da C.G. Diniz[a], MD, Aurimery G. Chermont[b], Ph.D., Kellen F.S. de Almeida[b], Ph.D, Salma B.Saraty[c], Ph.D., Mary L.F. M.F. de Mello[c], MD, Miriam R.C. Lima[c], MD, Patricia B. Carvalho[d], MD, Renata de B. Braga[d], MD, Kathia de O. Harada[d], MD, Maria C.A. Justino[e], Ph.D, Gleice Clemente[f], Ph.D, Maria Teresa Terreri[f, #], Ph.D, Marta C. Monteiro, Ph.D[g, #].

[#] These authors have contributed equally to this work as co-senior authorship.

CRITICAL MIS-C PATIENTS	SURVIVAL TIME IN DAYS	DEATH	RT-PCR TEST	ANTIGEN TEST	SEROLOGICAL TEST	SEX	ETHNICITY	AGE IN MONTHS	COMORBIDITIES	ORGAN DYSFUNCTION NUMBER
**1**	162	Alived	Negative	Positive	Negative	Female	Black	113,5	Yes	1
**2**	156	Alived	Negative	Positive	Negative	Female	Brazilian pardo	115,1	Yes	1
**3**	158	Alived	Negative	Positive	Negative	Female	Brazilian pardo	16,0	Yes	2
**4**	162	Alived	Negative	Positive	Negative	Female	Black	5,4	Yes	3
**5**	158	Alived	Negative	Positive	Negative	Female	White	114,8	Yes	4
**6**	163	Alived	Negative	Positive	Negative	Female	Black	114,8	Yes	1
**7**	161	Alived	Positve	Negative	Negative	Male	Brazilian pardo	24,5	Yes	4
**8**	4	Deceased	Negative	Positive	Positive	Male	Brazilian pardo	4,7	Yes	4
**9**	159	Alived	Negative	Positive	Positive	Male	Black	5,6	No	4
**10**	14	Deceased	Negative	Positive	Negative	Male	Black	23,0	Yes	4
**11**	162	Alived	Negative	Positive	Negative	Male	Black	107,8	No	1
**12**	162	Alived	Negative	Positive	Negative	Male	White	1,0	Yes	3
**13**	8	Deceased	Negative	Positive	Negative	Male	Black	20,2	Yes	4
**14**	202	Alived	Negative	Positive	Negative	Male	White	5,5	Yes	5
**15**	178	Alived	Negative	Positive	Negative	Male	White	14,6	Yes	1
**16**	3	Deceased	Positve	Negative	Negative	Male	Brazilian pardo	5,2	Yes	4
**17**	175	Alived	Negative	Positive	Negative	Male	Brazilian pardo	114,5	No	4
**18**	25	Deceased	Negative	Positive	Negative	Male	Asian	22,0	Yes	4
**19**	12	Deceased	Negative	Positive	Negative	Male	Brazilian pardo	23,0	Yes	4
**20**	11	Deceased	Negative	Positive	Negative	Male	Brazilian pardo	21,0	Yes	4
**21**	160	Alived	Negative	Positive	Positive	Male	Black	2,5	Yes	3
**22**	23	Deceased	Negative	Positive	Positive	Male	Brazilian pardo	5,3	Yes	5
**23**	165	Alived	Negative	Positive	Negative	Male	Brazilian pardo	112,7	Yes	1
**24**	6	Deceased	Negative	Positive	Negative	Male	Black	26,2	Yes	4
**25**	4	Deceased	Negative	Positive	Positive	Male	Black	25,1	Yes	4
**26**	19	Deceased	Positve	Negative	Negative	Male	Brazilian pardo	25,3	Yes	4
**27**	158	Alived	Positve	Negative	Negative	Male	Brazilian pardo	5,0	Yes	4

**28**	161	Alived	Positve	Negative	Negative	Male	Brazilian pardo	19,0	Yes	4
**29**	202	Alived	Negative	Positive	Positive	Male	Black	105,9	Yes	1
**30**	160	Alived	Negative	Positive	Positive	Male	Brazilian pardo	107,8	Yes	1
**31**	170	Alived	Negative	Positive	Positive	Male	Brazilian pardo	110,9	Yes	1
**32**	176	Alived	Negative	Positive	Negative	Male	Brazilian pardo	12,0	No	4
**33**	157	Alived	Positve	Negative	Negative	Male	Brazilian pardo	14,0	Yes	4
**34**	13	Deceas ed	Negative	Positive	Positive	Female	Brazilian pardo	25,4	Yes	4
**35**	163	Alived	Negative	Positive	Positive	Male	Brazilian pardo	115,5	Yes	1
**36**	162	Alived	Negative	Positive	Positive	Male	Brazilian pardo	116,0	Yes	1
**37**	159	Alived	Positve	Negative	Negative	Male	Brazilian pardo	13,7	Yes	4
**38**	6	Deceas ed	Negative	Positive	Negative	Male	Brazilian pardo	24,1	Yes	4
**39**	167	Alived	Negative	Positive	Positive	Male	White	129,0	Yes	1
**40**	165	Alived	Positve	Negative	Negative	Male	Brazilian pardo	110,2	Yes	1
**41**	7	Deceas ed	Negative	Positive	Negative	Male	Black	11,2	Yes	4
**42**	5	Deceas ed	Negative	Positive	Negative	Female	Brazilian pardo	3,5	Yes	4
**43**	165	Alived	Negative	Positive	Negative	Female	Brazilian pardo	109,2	Yes	3
**44**	170	Alived	Negative	Positive	Negative	Female	Black	23,0	No	1
**45**	162	Alived	Negative	Positive	Negative	Male	Brazilian pardo	113,0	No	1
**46**	173	Alived	Negative	Positive	Negative	Male	Brazilian pardo	125,8	No	1
**47**	171	Alived	Negative	Positive	Negative	Female	Asian	103,1	Yes	1
**48**	168	Alived	Negative	Positive	Negative	Female	Brazilian pardo	120,9	Yes	1
**49**	174	Alived	Positve	Negative	Negative	Female	Brazilian indian	26,0	Yes	3
**50**	158	Alived	Negative	Positive	Negative	Female	Black	107,0	Yes	2
**51**	161	Alived	Positve	Negative	Negative	Female	Asian	115,9	Yes	3
**52**	159	Alived	Negative	Positive	Positive	Male	Brazilian pardo	112,4	Yes	3
**53**	158	Alived	Negative	Positive	Positive	Male	Brazilian pardo	117,0	Yes	5
**54**	161	Alived	Negative	Positive	Positive	Male	Brazilian pardo	115,2	Yes	1
**55**	166	Alived	Negative	Positive	Negative	Male	Black	56,0	Yes	4
**56**	164	Alived	Positve	Negative	Negative	Male	Asian	115,8	Yes	6
**57**	158	Alived	Positve	Negative	Negative	Male	Brazilian pardo	114,1	Yes	3
**58**	161	Alived	Positve	Negative	Negative	Male	White	5,5	Yes	4

**59**	12	Deceased	Positve	Negative	Negative	Male	Asian	12,0	Yes	4
**60**	158	Alived	Positve	Negative	Negative	Male	Brazilian pardo	11,0	Yes	3
**61**	159	Alived	Positve	Negative	Negative	Male	Black	115,9	No	4
**62**	166	Alived	Positve	Negative	Negative	Male	Brazilian pardo	117,5	No	2
**63**	157	Alived	Positve	Negative	Negative	Male	Asian	5,5	Yes	3
**64**	169	Alived	Positve	Negative	Negative	Male	Brazilian pardo	5,5	No	3
**65**	157	Alived	Positve	Negative	Negative	Male	Black	5,2	No	2
**66**	165	Alived	Positve	Negative	Negative	Male	Black	5,1	No	4
**67**	169	Alived	Negative	Positive	Negative	Male	Brazilian pardo	5,3	Yes	1
**68**	160	Alived	Negative	Positive	Positive	Female	Brazilian pardo	5,2	Yes	3
**69**	168	Alived	Negative	Positive	Negative	Female	Brazilian pardo	5,1	Yes	2
**70**	182	Alived	Negative	Positive	Negative	Female	White	5,6	Yes	2
**71**	155	Alived	Negative	Positive	Negative	Female	White	5,7	Yes	2
**72**	22	Deceased	Negative	Positive	Negative	Male	Brazilian pardo	25,5	No	3
**73**	188	Alived	Negative	Positive	Negative	Male	White	5,0	Yes	3
**74**	185	Alived	Positve	Negative	Negative	Male	Asian	4,7	Yes	4
**75**	196	Alived	Negative	Positive	Negative	Female	Brazilian pardo	5,0	Yes	3
**76**	198	Alived	Negative	Positive	Negative	Female	Black	5,5	No	3
**77**	192	Alived	Negative	Positive	Negative	Male	Asian	5,2	No	2
**78**	184	Alived	Positve	Negative	Negative	Female	Brazilian indian	4,5	Yes	3
**79**	166	Alived	Negative	Positive	Negative	Female	Black	5,5	Yes	1
**SEVERE COVID-19 PATIENTS**	Survival time in days	Death	RT-PCR test	Antigen test	Serological test	Sex	Ethnicity	Age in months	Comorbidities	Organ dysfunction number
**1**	161	Alived	Positve	Negative	Not tested	Male	White	162,0	Yes	1
**2**	14	Deceased	Negative	Positive	Negative	Male	White	17,0	Yes	1
**3**	4	Deceased	Positve	Negative	Not tested	Male	White	3,0	Yes	1
**4**	161	Alived	Positve	Negative	Not tested	Female	Black	88,0	Yes	1
**5**	169	Alived	Positve	Negative	Not tested	Male	Brazilian pardo	48,0	No	1
**6**	162	Alived	Positve	Negative	Not tested	Female	White	2,0	No	1
**7**	161	Alived	Positve	Negative	Not tested	Female	White	26,0	Yes	1
**8**	169	Alived	Positve	Negative	Not tested	Male	White	48,0	Yes	1

**9**	169	Alived	Positve	Negative	Not tested	Female	Black	24,0	No	1
**10**	170	Alived	Negative	Positive	Not tested	Female	Black	26,0	No	1
**11**	162	Alived	Positve	Negative	Not tested	Female	Brazilian pardo	33,0	No	1
**12**	169	Alived	Positve	Negative	Not tested	Female	Black	6,0	No	1
**13**	169	Alived	Positve	Negative	Not tested	Female	Black	24,0	No	1
**14**	169	Alived	Negative	Positive	Not tested	Male	White	156,0	No	1
**15**	169	Alived	Positve	Negative	Not tested	Female	Black	11,0	No	1
**16**	170	Alived	Negative	Positive	Not tested	Male	White	12,0	Yes	1
**17**	169	Alived	Positve	Negative	Not tested	Female	Asian	77,0	Yes	1
**18**	162	Alived	Positve	Negative	Not tested	Female	Black	6,0	Yes	1
**19**	168	Alived	Positve	Negative	Not tested	Male	Brazilian indian	1,0	No	1
**20**	169	Alived	Negative	Positive	Not tested	Female	White	52,0	No	1
**21**	169	Alived	Negative	Positive	Not tested	Female	White	30,0	No	1
**22**	162	Alived	Negative	Positive	Not tested	Male	Brazilian indian	1,0	No	1
**23**	170	Alived	Negative	Positive	Not tested	Male	White	18,5	No	1
**24**	169	Alived	Positve	Negative	Not tested	Female	Black	12,0	No	1
**25**	160	Alived	Positve	Negative	Not tested	Male	White	83,0	Yes	1
**26**	161	Alived	Positve	Negative	Not tested	Female	Brazilian pardo	18,0	No	1
**27**	153	Alived	Negative	Positive	Not tested	Male	Brazilian pardo	23,0	No	1
**28**	9	Deceased	Negative	Positive	Not tested	Male	White	27,0	No	1
**29**	168	Alived	Negative	Positive	Negative	Female	Brazilian pardo	4,7	Yes	4
**30**	162	Alived	Negative	Positive	Negative	Male	Brazilian pardo	5,0	Yes	1
**31**	13	Deceased	Negative	Positive	Not tested	Male	Black	4,0	No	1
**32**	161	Alived	Negative	Positive	Negative	Female	Brazilian pardo	6,0	No	1
**33**	155	Alived	Negative	Positive	Negative	Female	Black	49,0	No	1
**34**	153	Alived	Negative	Positive	Negative	Female	Brazilian pardo	2,0	Yes	1
**35**	6	Deceased	Negative	Positive	Negative	Male	White	141,0	Yes	1
**36**	5	Deceased	Positve	Negative	Not tested	Male	White	86,5	Yes	1
**37**	155	Alived	Negative	Positive	Negative	Male	Black	4,8	No	1
**38**	4	Deceased	Positve	Negative	Not tested	Male	White	51,0	Yes	1

**39**	152	Alived	Negative	Positive	Negative	Male	Brazilian pardo	128,0	No	1
**40**	152	Alived	Negative	Positive	Negative	Male	Brazilian pardo	4,0	No	1
**41**	9	Deceas ed	Positve	Negative	Not tested	Male	White	147,0	Yes	1
**42**	169	Alived	Negative	Positive	Not tested	Male	White	13,5	Yes	1
**43**	170	Alived	Negative	Positive	Not tested	Female	Brazilian pardo	75,0	Yes	1
**44**	169	Alived	Negative	Positive	Not tested	Female	Brazilian pardo	163,0	Yes	1
**45**	169	Alived	Negative	Positive	Not tested	Female	Black	73,4	Yes	1
**46**	153	Alived	Negative	Positive	Not tested	Male	Brazilian pardo	169,3	Yes	3
**47**	169	Alived	Negative	Positive	Not tested	Male	Brazilian pardo	21,2	No	1
**48**	169	Alived	Negative	Positive	Not tested	Male	Brazilian pardo	80,5	Yes	1
**49**	169	Alived	Negative	Positive	Not tested	Male	Brazilian pardo	5,5	No	1
**50**	170	Alived	Negative	Positive	Positive	Female	Brazilian pardo	21,6	No	1
**51**	162	Alived	Positve	Negative	Not tested	Female	Brazilian pardo	80,5	No	1
**52**	153	Alived	Negative	Positive	Not tested	Female	Brazilian pardo	4,9	No	1
**53**	155	Alived	Positve	Negative	Not tested	Female	Brazilian pardo	13,7	No	1
**54**	171	Alived	Negative	Positive	Not tested	Male	White	89,0	No	1
**55**	169	Alived	Positve	Negative	Not tested	Male	Brazilian indian	86,3	No	1
**56**	153	Alived	Positve	Negative	Not tested	Female	Brazilian pardo	19,8	Yes	1
**57**	153	Alived	Positve	Negative	Not tested	Male	Brazilian pardo	28,3	Yes	1
**58**	154	Alived	Negative	Positive	Not tested	Male	Brazilian pardo	5,1	Yes	1
**59**	168	Alived	Negative	Positive	Not tested	Female	Brazilian indian	21,8	No	1
**60**	169	Alived	Negative	Positive	Not tested	Female	Brazilian pardo	2,9	No	1
**61**	170	Alived	Positve	Negative	Not tested	Male	White	180,7	Yes	1
**62**	170	Alived	Positve	Negative	Not tested	Male	White	18,1	No	1
**63**	170	Alived	Negative	Positive	Positive	Female	White	5,2	No	1
**64**	169	Alived	Positve	Negative	Not tested	Male	White	2,7	No	1
**65**	154	Alived	Positve	Negative	Not tested	Female	White	1,4	No	1
**66**	170	Alived	Positve	Negative	Not tested	Male	White	1,4	No	1
**67**	153	Alived	Negative	Positive	Positive	Female	Brazilian pardo	135,9	No	1
**68**	169	Alived	Positve	Negative	Not tested	Female	Brazilian pardo	15,5	No	1
**69**	154	Alived	Negative	Positive	Not tested	Male	Brazilian pardo	15,0	No	1
**70**	163	Alived	Negative	Positive	Positive	Female	Brazilian pardo	132,6	No	1
**71**	169	Alived	Negative	Positive	Positive	Female	Black	142,7	No	1

**72**	169	Alived	Negative	Positive	Positive	Female	Brazilian pardo	28,0	No	1
**73**	171	Alived	Positve	Negative	Not tested	Male	Brazilian pardo	0,9	No	1
**74**	162	Alived	Positve	Negative	Not tested	Male	White	5,2	No	1
**75**	163	Alived	Negative	Positive	Not tested	Female	Brazilian pardo	2,2	Yes	1
**76**	163	Alived	Negative	Positive	Not tested	Male	Brazilian pardo	80,4	Yes	1
**77**	171	Alived	Negative	Positive	Positive	Female	Brazilian pardo	3,0	Yes	1
**78**	6	Deceas ed	Negative	Positive	Not tested	Male	Brazilian pardo	18,4	Yes	1
**79**	164	Alived	Negative	Positive	Not tested	Female	Brazilian pardo	129,3	Yes	3
**80**	152	Alived	Negative	Positive	Not tested	Female	Brazilian pardo	4,6	Yes	3
**81**	27	Deceas ed	Negative	Positive	Not tested	Male	White	120,1	Yes	1
**82**	155	Alived	Negative	Positive	Not tested	Female	Brazilian pardo	3,3	Yes	1
**83**	153	Alived	Negative	Positive	Not tested	Female	Brazilian pardo	2,9	Yes	1
**84**	163	Alived	Negative	Positive	Not tested	Female	Brazilian pardo	74,8	Yes	3
**85**	14	Deceas ed	Negative	Positive	Not tested	Male	Black	31,1	Yes	3
**86**	163	Alived	Negative	Positive	Not tested	Female	White	14,8	No	3
**87**	169	Alived	Negative	Positive	Not tested	Female	Black	73,4	Yes	1
**88**	169	Alived	Negative	Positive	Not tested	Female	Black	83,5	Yes	2
**89**	152	Alived	Negative	Positive	Not tested	Female	Brazilian pardo	4,4	Yes	3
**90**	2	Deceas ed	Negative	Positive	Not tested	Male	White	80,8	Yes	1
**91**	153	Alived	Negative	Positive	Not tested	Female	White	5,2	Yes	1
**92**	4	Deceas ed	Negative	Positive	Not tested	Female	White	169,3	Yes	3
**93**	162	Alived	Negative	Positive	Not tested	Female	Brazilian pardo	19,5	Yes	3
**94**	162	Alived	Negative	Positive	Not tested	Female	White	163,0	Yes	1
**95**	163	Alived	Negative	Positive	Not tested	Female	Brazilian pardo	13,0	Yes	1
**96**	163	Alived	Negative	Positive	Not tested	Female	Brazilian pardo	159,3	Yes	1
**97**	2	Deceas ed	Negative	Positive	Not tested	Female	White	84,7	Yes	4
**98**	163	Alived	Negative	Positive	Not tested	Female	Brazilian indian	21,4	No	1
**99**	163	Alived	Negative	Positive	Not tested	Female	Black	5,5	No	1
**100**	164	Alived	Negative	Positive	Not tested	Female	Brazilian pardo	24,6	Yes	1

**101**	3	Deceased	Positve	Negative	Not tested	Female	Black	81,5	Yes	2
**102**	162	Alived	Positve	Negative	Not tested	Female	White	5,6	Yes	1
**103**	162	Alived	Negative	Positive	Not tested	Female	Black	53,9	No	1
**104**	161	Alived	Positve	Negative	Not tested	Female	Brazilian pardo	6,2	No	1
**105**	7	Deceased	Negative	Positive	Positive	Female	Black	67,6	Yes	2
**106**	14	Deceased	Negative	Positive	Positive	Female	Brazilian pardo	72,8	Yes	4
**107**	169	Alived	Negative	Positive	Positive	Male	Brazilian pardo	28,9	Yes	1
**108**	160	Alived	Negative	Positive	Not tested	Male	Brazilian pardo	10,1	Yes	1
**109**	3	Deceased	Negative	Positive	Not tested	Male	Black	143,8	Yes	6
**110**	169	Alived	Positve	Negative	Not tested	Male	Brazilian pardo	17,0	No	1
**111**	171	Alived	Positve	Negative	Not tested	Male	Brazilian pardo	74,1	No	1
**112**	163	Alived	Positve	Negative	Not tested	Female	Brazilian pardo	91,6	No	1
**113**	307	Alived	Positve	Negative	Not tested	Male	Brazilian pardo	81,5	No	1
**114**	169	Alived	Positve	Negative	Not tested	Male	Black	28,0	No	1
**115**	163	Alived	Positve	Negative	Not tested	Female	Brazilian pardo	27,9	No	1
**116**	162	Alived	Positve	Negative	Not tested	Female	White	26,4	No	1
**117**	161	Alived	Positve	Negative	Not tested	Female	Asian	82,9	Yes	1
**118**	6	Deceased	Positve	Negative	Not tested	Male	Brazilian pardo	4,0	Yes	4
**119**	172	Alived	Positve	Negative	Not tested	Male	Black	20,3	Yes	1
**120**	169	Alived	Negative	Positive	Not tested	Male	Brazilian pardo	12,4	Yes	1
**121**	12	Deceased	Positve	Negative	Not tested	Female	Black	5,3	Yes	1
**122**	155	Alived	Negative	Positive	Not tested	Male	Brazilian pardo	28,3	Yes	1
**123**	153	Alived	Negative	Positive	Not tested	Male	Brazilian pardo	131,7	Yes	1
**124**	160	Alived	Negative	Positive	Not tested	Male	Brazilian pardo	4,6	Yes	1
**125**	153	Alived	Negative	Positive	Not tested	Male	Brazilian indian	5,2	Yes	1
**126**	170	Alived	Negative	Positive	Not tested	Male	Brazilian pardo	2,1	Yes	1
**127**	170	Alived	Negative	Positive	Not tested	Male	Brazilian pardo	22,4	Yes	1
**128**	169	Alived	Positve	Negative	Not tested	Male	White	82,0	No	1
**129**	169	Alived	Positve	Negative	Not tested	Male	Black	80,7	Yes	1
**130**	169	Alived	Positve	Negative	Not tested	Male	Brazilian pardo	13,7	No	1

**131**	157	Alived	Positve	Negative	Not tested	Male	White	1,4	Yes	1
**132**	164	Alived	Positve	Negative	Not tested	Female	Brazilian indian	19,8	Yes	1
**133**	162	Alived	Positve	Negative	Not tested	Female	White	52,4	Yes	1
**134**	152	Alived	Positve	Negative	Not tested	Male	Brazilian pardo	16,0	Yes	1
**135**	153	Alived	Negative	Positive	Positive	Male	Brazilian pardo	21,6	No	2
**136**	162	Alived	Negative	Positive	Not tested	Male	Brazilian pardo	16,3	Yes	3
**137**	2	Deceased	Positve	Negative	Not tested	Male	Black	140,4	Yes	1
**138**	4	Deceased	Positve	Negative	Not tested	Male	White	3,0	Yes	2
**139**	28	Deceased	Negative	Positive	Negative	Female	White	23,0	No	2
**140**	26	Deceased	Negative	Positive	Negative	Male	White	24,2	No	2
**141**	5	Deceased	Negative	Positive	Negative	Male	White	111,0	No	2
**142**	9	Deceased	Negative	Positive	Not tested	Male	Brazilian pardo	23,1	No	2
**143**	5	Deceased	Negative	Positive	Negative	Male	White	80,8	No	1
**144**	13	Deceased	Negative	Positive	Not tested	Male	Brazilian pardo	4,0	No	1
**145**	5	Deceased	Positve	Negative	Not tested	Female	White	88,0	Yes	1
**146**	4	Deceased	Positve	Negative	Not tested	Male	Black	51,0	No	1
**147**	9	Deceased	Positve	Negative	Not tested	Female	White	147,0	Yes	1
**148**	25	Deceased	Negative	Positive	Negative	Male	White	6,0	Yes	1
**149**	12	Deceased	Negative	Positive	Negative	Male	White	3,0	Yes	1
**150**	14	Deceased	Negative	Positive	Negative	Female	Black	132,0	No	1
**151**	14	Deceased	Negative	Positive	Negative	Male	Black	3,0	Yes	1
**152**	3	Deceased	Negative	Positive	Negative	Male	Brazilian pardo	12,0	Yes	1
**153**	6	Deceased	Negative	Positive	Not tested	Male	Brazilian pardo	18,4	Yes	1

**154**	24	Deceased	Negative	Positive	Not tested	Male	Black	120,1	Yes	1
**155**	14	Deceased	Negative	Positive	Not tested	Male	White	31,1	Yes	1
**156**	2	Deceased	Negative	Positive	Not tested	Male	Brazilian pardo	76,8	Yes	1
**157**	4	Deceased	Negative	Positive	Not tested	Female	Brazilian pardo	169,3	Yes	1
**158**	2	Deceased	Negative	Positive	Not tested	Female	Brazilian pardo	74,7	Yes	1
**159**	154	Alived	Positve	Negative	Not tested	Female	White	6,2	No	3
**160**	153	Alived	Negative	Positive	Positive	Male	White	28,9	Yes	1
**161**	161	Alived	Negative	Positive	Not tested	Male	White	10,1	Yes	1
**162**	162	Alived	Positve	Negative	Not tested	Male	Black	17,0	No	1
**163**	152	Alived	Positve	Negative	Not tested	Male	Black	74,1	No	1
**164**	164	Alived	Positve	Negative	Not tested	Female	Black	91,6	No	2
**165**	162	Alived	Positve	Negative	Not tested	Male	Asian	74,9	No	3
**166**	170	Alived	Positve	Negative	Not tested	Male	Brazilian pardo	28,0	No	2
**167**	163	Alived	Positve	Negative	Not tested	Male	Asian	27,9	No	3
**168**	161	Alived	Positve	Negative	Not tested	Male	Asian	22,6	Yes	1
**169**	160	Alived	Positve	Negative	Not tested	Male	Brazilian pardo	118,7	Yes	1
**170**	155	Alived	Positve	Negative	Not tested	Male	Brazilian indian	20,3	Yes	3
**171**	161	Alived	Negative	Positive	Not tested	Male	Brazilian indian	12,4	Yes	1
**172**	162	Alived	Negative	Positive	Not tested	Male	Brazilian indian	28,3	Yes	3
**173**	171	Alived	Negative	Positive	Not tested	Male	Brazilian indian	131,7	Yes	4
**174**	171	Alived	Negative	Positive	Not tested	Male	Asian	4,6	Yes	5
**175**	162	Alived	Negative	Positive	Not tested	Male	Brazilian pardo	5,2	Yes	6
**176**	153	Alived	Negative	Positive	Not tested	Male	White	2,1	Yes	3
**177**	153	Alived	Negative	Positive	Not tested	Male	White	22,2	Yes	4
**178**	152	Alived	Positve	Negative	Not tested	Male	White	82,0	No	1
**179**	153	Alived	Positve	Negative	Not tested	Male	Brazilian pardo	80,6	Yes	1
**180**	155	Alived	Positve	Negative	Not tested	Male	Black	13,7	Yes	1
**181**	155	Alived	Positve	Negative	Not tested	Male	Black	1,4	Yes	2
**182**	163	Alived	Positve	Negative	Not tested	Male	Brazilian pardo	19,8	Yes	1
**183**	156	Alived	Positve	Negative	Not tested	Male	Black	52,4	Yes	1

**184**	153	Alived	Positve	Negative	Not tested	Male	Black	16,0	Yes	1
**185**	169	Alived	Negative	Positive	Positive	Male	Brazilian pardo	24,1	No	1
**186**	154	Alived	Negative	Positive	Not tested	Male	Brazilian indian	16,3	Yes	3
**187**	169	Alived	Positve	Negative	Not tested	Female	Brazilian pardo	24,1	Yes	4
